# Visualizing electronic interactions between iron and carbon by X-ray chemical imaging and spectroscopy†
†Electronic supplementary information (ESI) available: Experimental details, computational details and Fig. S1–S10. See DOI: 10.1039/c5sc00353a



**DOI:** 10.1039/c5sc00353a

**Published:** 2015-03-26

**Authors:** Xiaoqi Chen, Jianping Xiao, Jian Wang, Dehui Deng, Yongfeng Hu, Jigang Zhou, Liang Yu, Thomas Heine, Xiulian Pan, Xinhe Bao

**Affiliations:** a State Key Laboratory of Catalysis , iChEM , Dalian Institute of Chemical Physics , Chinese Academy of Sciences , Zhongshan Road 457 , Dalian , 116023 , China . Email: dhdeng@dicp.ac.cn ; Email: xhbao@dicp.ac.cn ; Fax: +86-411-84379128 ; Tel: +86-411-84686637; b Canadian Light Source Inc. , University of Saskatchewan , 44 Innovation Boulevard , Saskatoon , SK S7N 2V3 , Canada; c Department of Physics and Earth Science , Jacobs University Bremen , Campus Ring 1 , 28759 Bremen , Germany

## Abstract

The electronic interaction of a catalyst and its support is of vital importance to its catalytic performance. However, it is still a great challenge to directly probe the interaction due to the lack of well-defined models and efficient technical means. In this study, we report that pod-like carbon nanotubes with encapsulated iron particles (Pod-Fe) and scanning transmission X-ray microscopy (STXM) can be used as an ideal model and technique to study the electronic interaction between carbon shells and iron particles. The chemical imaging and spectroscopy of Pod-Fe by STXM show that the local electronic structures at C K-edge near edge (π*) of carbon shells can be significantly modified by the encapsulated iron particles, which promotes the adsorption of oxygen-containing species, and thereby further modifies the electronic structure (π* and σ*) of the carbon shells. Moreover, computed X-ray absorption near edge structure spectra (XANES) confirmed the electronic modifications of carbon shells by the encapsulated iron particles. The present study provides a direct evidence of electronic interactions with simultaneously collected images and spectra, which can promote the understanding towards the nature of active sites and supports.

## Abstract 2

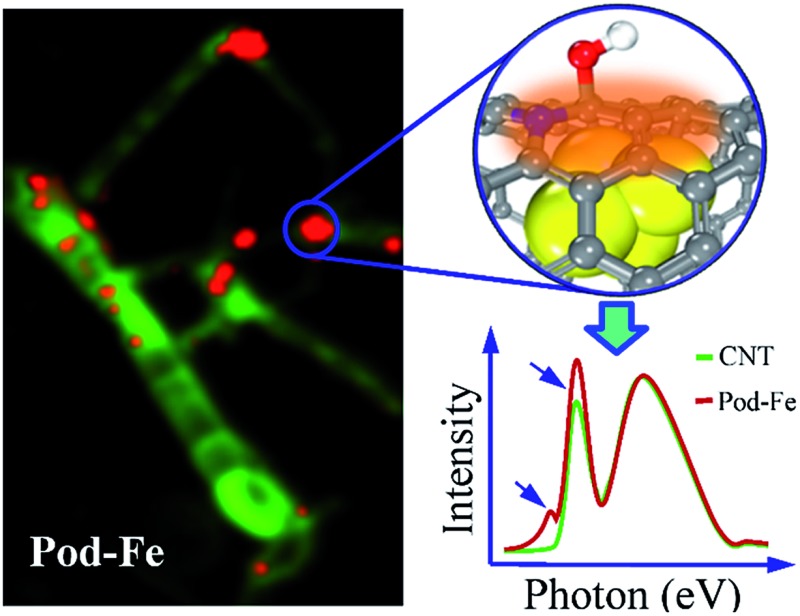
Pod-like carbon nanotube with encapsulated iron particles (Pod-Fe) was used as a well-defined model to study the electronic interaction between carbon shells and the iron particles by scanning transmission X-ray microscopy (STXM).

## Introduction

Tunable performance of a supported catalyst is often ascribed to the strong interaction between the active component and its support.^1,2^ The interaction between the active sites with support material is, therefore, of great importance and thus has been the subject of intensive studies in electro- and heterogeneous catalysis.^3–6^ A number of recent works by our and other groups have indicated that there is a strong electronic interaction between carbon shells and encapsulated transition metals (such as Fe, Co, Ni, and their alloys), for example, in the case of the pod-like carbon nanotubes with encapsulated iron particles (Pod-Fe),^7^ thereby tuning the activity on the carbon shells in various reactions such as oxygen reduction reaction (ORR) in fuel cells,^7–10^ hydrogen evolution reaction (HER),^11–13^ I_3_^–^ reduction reaction in dye-sensitized solar cells (DSSCs),^14^ as well as catalytic oxidation and reduction reactions in heterogeneous catalysis.^15,16^ Though density functional calculations have indicated the electronic structures of carbon shells could be modified by the encapsulated metals, direct experimental evidence for this interaction is still lacking.^7,17^ It is quite difficult to directly observe the interaction because many technical means cannot image a complex system and simultaneously collect spectroscopic signals with both high energy and spatial resolution. For example, some spectroscopic techniques such as X-ray absorption near edge structure (XANES) and X-ray photoelectron spectroscopy (XPS) have high energy resolution, but the signals of samples are always overlapped by a wide range of localized electronic information due to their low spatial resolution.^18^ For the experimental techniques with high spatial resolution imaging such as transmission electron microscopy (TEM) and scanning electron microscopy (SEM) combined with electron energy-loss spectroscopy (EELS), the practical use is limited due to high radiation damage of the exposed probe materials.^19,20^ Scanning tunneling microscopy and scanning tunneling spectroscopy (STM/STS) can provide both high resolution images and electronic structure information, but they are only sensitive to surface states and require an atomically-flat sample surface. Hence, it is difficult to apply electron microscopy for real catalysts.^21,22^ Compared with the above techniques, scanning transmission X-ray microscopy (STXM), a synchrotron-based spectromicroscopic technique, is a very effective and versatile tool for simultaneous chemical imaging and spectroscopy of samples at nano- and sub-micron scales.^23–27^ It provides both high spatial resolution (<30 nm) and high energy resolution (<0.1 eV). Herein, by using a combination of the STXM imaging technique with state-of-the-art X-ray absorption spectral calculations, we have investigated the electronic states of carbon nanotubes with encapsulated iron nanoparticles. The local electronic structure variations on carbon shells induced by the encapsulated iron nanoparticles are successfully captured by STXM, and in combination with the computed X-ray absorption spectra, we have interpreted the chemical origin of the electronic structure variations. The present study provides direct imaging, spectral evidence and theoretical support for the strong interactions of carbon shells with encapsulated iron nanoparticles.

## Results and discussion

The metallic iron encapsulated pod-like carbon nanotubes (Pod-Fe) were synthesized *via* heat-treating (NH_4_)_4_Fe(CN)_6_ at 600 °C followed by an acid washing process to remove the exposed iron particles according to our previous report.^7^ STXM imaging and spectroscopy were performed on the Soft X-ray Spectromicroscopy beamline (SM) 10ID-1 at the Canadian Light Source (CLS). XANES spectra at the K-edges of C, N and O and on the Fe L-edge were extracted from STXM image stacks that were obtained by scanning over a range of photon energies. XANES spectra calculations were carried out based on the multiple scattering scheme, as implemented in FEFF9.0 code (see more details in ESI†).^28^

**Fig. 1:**
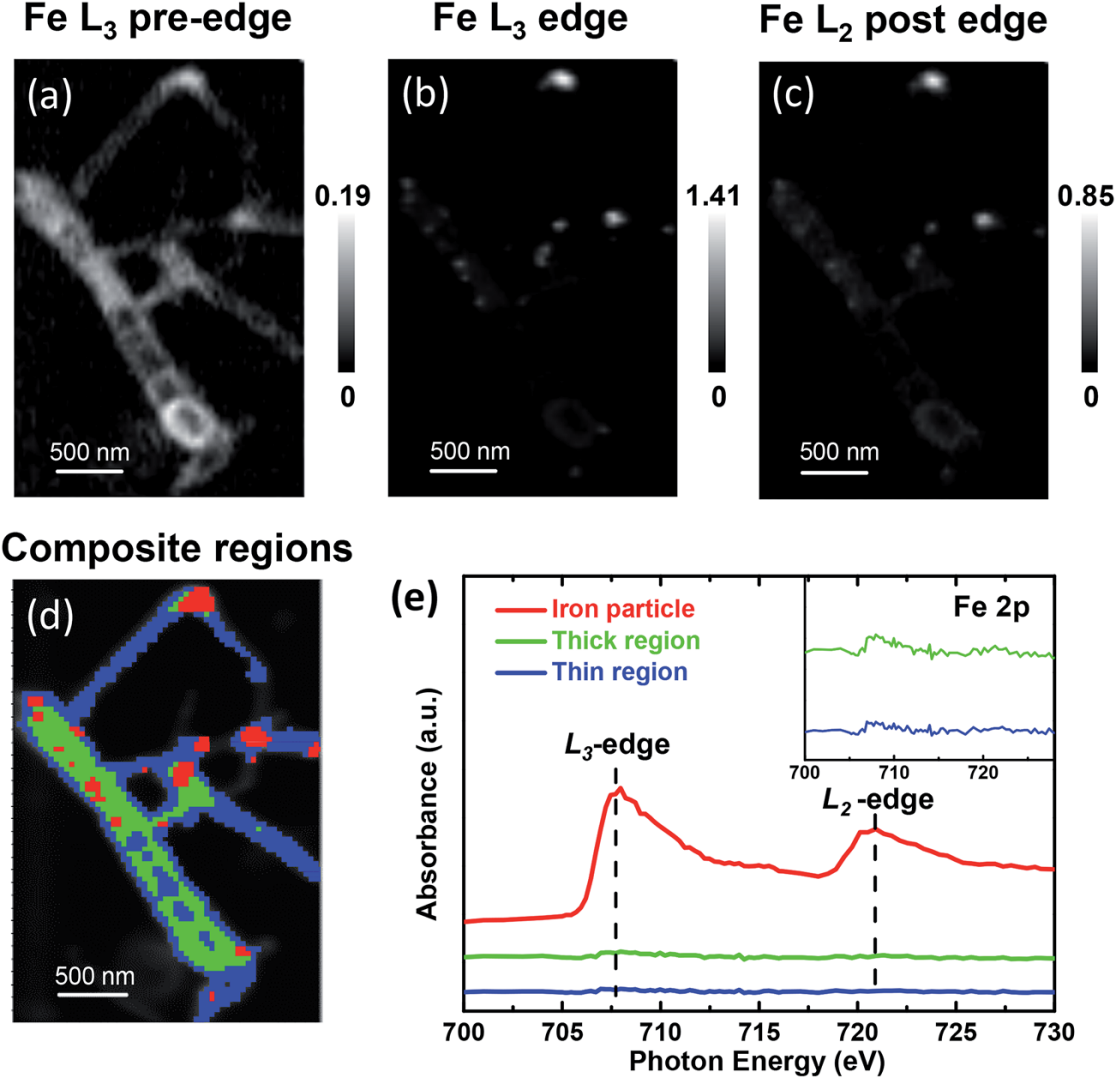
STXM chemical images of the pod-like carbon nanotube with encapsulated iron (Pod-Fe). High-resolution STXM transmission images at (a) Fe L_3_ pre-edge (700.0 eV). (b) Fe L_3_ edge (708.0 eV) and (c) Fe L_2_ post-edge (728.0 eV). The scale bars on the right represent the absorbance intensity. (d) Color composite regions, red: iron particle regions; green: thick CNT regions; blue: thin CNT regions. (e) Fe L-edge spectra of the corresponding three regions in (d). The inset shows the iron signal of thick and thin regions in expanded scale.

The STXM chemical images of the pod-like carbon nanotube with encapsulated iron particles at Fe L_3_ pre-edge (700.0 eV), L_3_ edge (708.0 eV), and L_2_ post-edge (728.0 eV) by STXM are shown in Fig. 1a–c, respectively, which is further confirmed by TEM image (Fig. S1†). At the Fe L_3_ pre-edge (Fig. 1a), only the morphology of the carbon nanotube (CNT) can be seen since the incident photon energy is not high enough to excite Fe 2p electrons. When the energy increased to the Fe L_3_ edge and Fe L_2_ post-edge, some white dots appear in the CNT due to the strong X-ray absorption at the edges, which originate from the encapsulated metallic iron nanoparticles. Based on the contrast of the stack images, the sample images could be roughly divided into three regions (segmentation): iron particle regions, thick CNT regions, and thin CNT regions, as marked by red, green and blue, respectively. The color composite regions of the sample are shown in Fig. 1d, and the corresponding Fe spectra from the three regions are shown in Fig. 1e. Obviously, one can see that there are almost no signals of iron nanoparticles at the thick and thin CNT regions (green and blue lines in Fig. 1e), while the iron particle regions primarily show a metallic Fe signal (red line in Fig. 1e). Note these iron nanoparticles are completely encapsulated in the compartments of the CNTs because the outside iron nanoparticles have been removed by thorough acid washing, as shown in the Experimental section in the ESI† and in our previous report.^7^ The Fe L-edge spectra of the powder sample measured by fluorescence yield mode (FY) indicate the iron state of the powder sample is metallic. The total electron yield (TEY), which was measured simultaneously with FY, shows a weak shoulder peak at L_3_ edge, indicating there is electron transfer from iron to CNT (Fig. S2†). Although TEY is a more surface-sensitive technique, the characteristic splitting at L_2_ edge of Fe oxides found in the work of Regan *et al.* ^29^ was not observed in our TEY mode. Instead, the DFT calculations in our previous work indicates there is electron transfer from iron to carbon for the Pod-Fe sample.^7^ Therefore, the trace electron loss of iron is probably due to the interaction between iron and carbon, instead of oxidation.

**Fig. 2:**
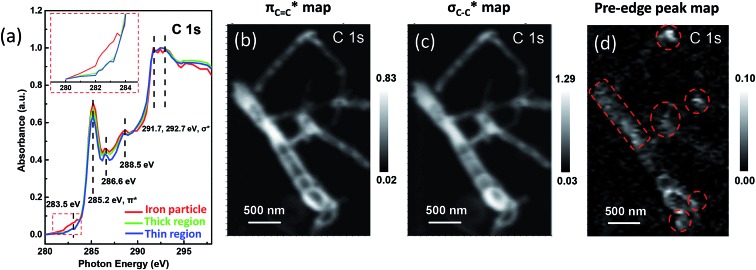
STXM XANES spectra and mappings of C 1s on Pod-Fe. (a) Selected regions on the sample (Fig. 1d) to extract normalized C 1s XANES spectra from the STXM stack (normalization range from the lowest absorbance to the highest), the inset shows the magnified image of the dashed red rectangle. (b) π_C=C_* bond map, using the π_C=C_* averaged images (from 285 to 285.5 eV) subtracting the pre-edge averaged images (from 280 to 282 eV). (c) σ_C–C_* bond map, using the σ_C–C_* averaged images (from 291.5 to 292.5 eV) subtracting the pre-edge averaged images (from 280 to 282 eV). (d) Pre-edge peak map at 283.5 eV, using the pre-edge peak averaged images (from 282.5 to 283.5 eV) subtracting the pre-edge averaged images (from 280 to 281 eV). The red dashed circles indicate the main iron particles' position. The scale bars on the right represent the absorbance intensity.

**Fig. 3:**
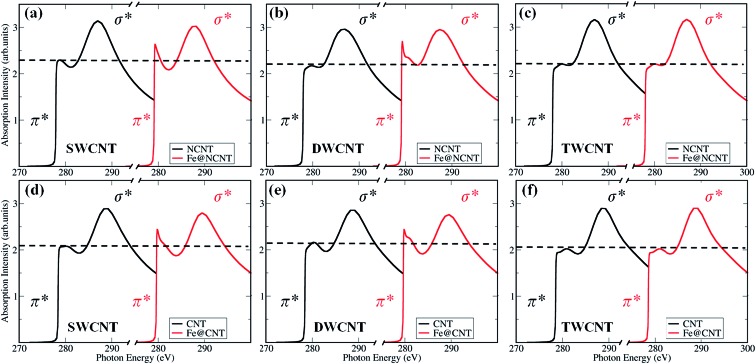
Calculated X-ray absorption spectra of carbon atoms at K-edge on CNTs with and without Fe_4_ cluster (see Fig. S8 in ESI† for calculation details and models). (a) A single-walled CNT (SWCNT), (b) a double-walled CNT (DWCNT), and (c) a triple-walled CNT (TWCNT) with a nitrogen dopant in the vicinity of the probed carbon atom; and undoped (d) SWCNT, (e) DWCNT, and, (f) TWCNT, respectively.

In order to explore the electronic interactions of carbon with the encapsulated iron particles, we analyzed the electronic structure of the carbon shells. It is generally known that the spectral features at ∼285 and ∼292 eV are attributed to the transitions from C 1s to graphitic states of π* and σ*, respectively (Fig. 2a). The most intense peaks at about 291.7 and 292.7 eV were featured as a resolved double-peak due to σ_C–C_* resonance, confirming that the three regions of samples are highly graphitized, which is also reflected in XANES spectra of powder samples (Fig. S3†).^30,31^ Moreover, we found that the C 1s π* signals (285.2 eV) in the spectrum of iron particle regions (Fig. 2a and S4†) are higher compared with those in the thin and thick CNT regions. Hence, we performed XANES calculations to elucidate the enhanced π* signals of carbon shells, as shown in Fig. 3. One can see the X-ray absorption intensity of π* bonds of a single-walled CNT (SWCNT) with encapsulated iron increases significantly compared with that of pure SWCNT (Fig. 3d), which is in good agreement with the experimental results. In addition the CNT sample was doped with nitrogen in experiments in order to improve the catalytic activity (Fig. S5†),^7^ we, therefore, considered nitrogen-doped CNT (NCNT) in spectral calculations too. The trend is quite similar in the case of a NCNT system, *i.e.* the π* features of carbon are always enhanced at the iron cluster regions (Fig. 3a). This is due to the hybridization of unoccupied 3d orbitals from encapsulated iron particles with carbon/nitrogen 2p orbitals, consequently enhancing the intensity of π* signals. In addition, we have also observed a similar character for a double-walled CNT (Fig. 3b and e). By further increasing the thickness of the CNT, the variation in π* bond intensity almost vanishes, as shown in Fig. 3c and f, suggesting that the effects on the outer carbon from encapsulated iron become weaker for a multi-walled CNT. As the size of iron particles in the Pod-Fe samples is much larger compared with those of the iron cluster in our calculations, the electronic effects from encapsulated iron particles should be extended to more carbon shells in the Pod-Fe samples. Note that the absolute energy position of σ* and π* bonds in computed spectra is about 4 eV lower than that in experiments. This is most likely due to our small simulation model and local density approximation in spectrum calculations, and this systematic shift can be corrected in the analysis. However, the main spectral features in experiments have been captured by our calculations.

Moreover, we investigated the spatial distribution of the carbon π* and σ* bonds by mapping the different bonds using the image stacks subtracted from pre-edge. For example, the mapping of π_C=C_* edge in Fig. 2b was derived from the averaged images (from 285 to 285.5 eV) subtracting the averaged images (from 280 to 282 eV). The π_C=C_* and σ_C–C_* map clearly shows the pod-like morphology of the carbon nanotube (Fig. 2b and c). Most strikingly, when looking into the pre-edge peak mapping, *i.e.*, the signal at ∼283.5 eV, it is clear that there are white dots showing higher optical density (Fig. 2d). The white dots in the map are at the same locations as the iron particles which are marked by red dashed circles, confirming that the pre-edge signal is associated with the iron nanoparticles. Therefore, the interaction between iron and carbon is clearly evident.

Previous studies also reported that the pre-edge peak has a close correlation with the electronic structure variation of carbon.^32–36^ For example, in the system of Ba or FeCl_3_ intercalated single-walled carbon nanotubes (SWCNTs), the presence of pre-edge peaks was attributed to the hybridization of Ba or FeCl_3_ with SWCNTs.^33,34^ Strong covalent metal-d–graphene-π hybridization is also observed upon deposition of Ni and Co metal contacts onto graphene/SiO_2_, which shows a low-energy shoulder to the π^*^ feature.^35^ Iyer *et al.* also found that the pre-edge of carbon can emerge after the annealing of graphene-supported Au particles due to the charge transfer between Au and graphene.^36^ Although the signal of the pre-edge was ascribed to the interaction between the metal and the carbon, the chemical origin is still unclear. In our previous work, DFT calculations indicated that the encapsulated iron particles are able to improve the adsorption of oxygen-containing species on the carbon surface,^7^ and enhance the ORR activity.^8–10^ In this case, the interaction between iron and carbon will play an important role in the adsorption of oxygen-containing species, which should be reflected by the spectra of Pod-Fe.

**Fig. 4:**
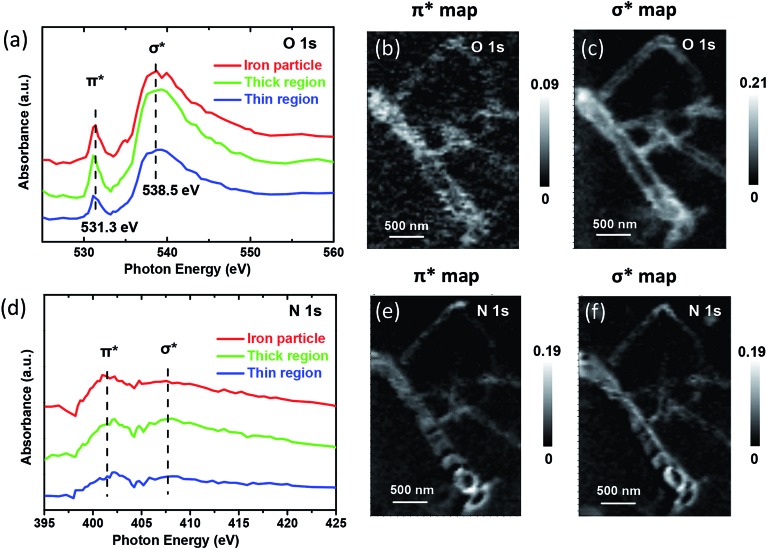
STXM XANES spectra and mappings of O 1s and N 1s on Pod-Fe. (a) Selected regions on the sample (Fig. 1d) to extract O K-edge XANES spectra from the STXM stacks. (b) O 1s π* bond map and (c) σ* bond map of Pod-Fe, using the π* averaged images (from 531 to 531.5 eV) and σ* averaged images (from 537.5 to 540.5 eV) subtracting the pre-edge averaged images (from 526 to 528 eV), respectively. (d) N K-edge XANES spectra extracted from the STXM stacks. (e) N 1s π* bond map and (f) σ* bond map, using the π* averaged images (from 401.7 to 402.7 eV) and σ* averaged images (from 407.5 to 408.5 eV) subtracting the pre-edge averaged images (from 395 to 397 eV), respectively. The scale bars on the right represent the absorbance intensity.

Indeed, there are two weak signals at 286.6 and 288.5 eV in C K-edge XANES, attributed to the electronic states (π_C=O_* or σ_C–O_*) of oxygen-containing species,^37,38^ indicating the possible presence of oxygen-containing species on the CNT samples. This oxygen-containing species should originate from the adsorption of the oxygen in the air after the sample synthesis. This was further confirmed by significant signals of adsorbed oxygen-containing species on the CNT samples as shown in Fig. 4a and Fig. S6,† in which the peaks at about 531.3 and 538.5 eV correspond to O 1s π* and σ* bond, respectively. From the chemical maps generated for these species, it can be clearly seen that the O 1s bond maps (Fig. 4b and c) only show the tube walls, while the compartment walls cannot be observed in contrast to the bond maps of C 1s (Fig. 2b and c) and N 1s (Fig. 4e and f). It indicates that the oxygen-containing species are mainly distributed on the exterior of the CNTs. The estimated elemental composition also confirmed that the thin region of CNTs which is related with exterior of CNTs has higher oxygen density (0.37) than thick region (0.23) relative to carbon (Table S1†). More interestingly, the iron particle region has a higher oxygen density (0.45) compared with thin and thick regions, which indicates the iron particles promote the adsorption of oxygen species on CNTs.

**Fig. 5:**
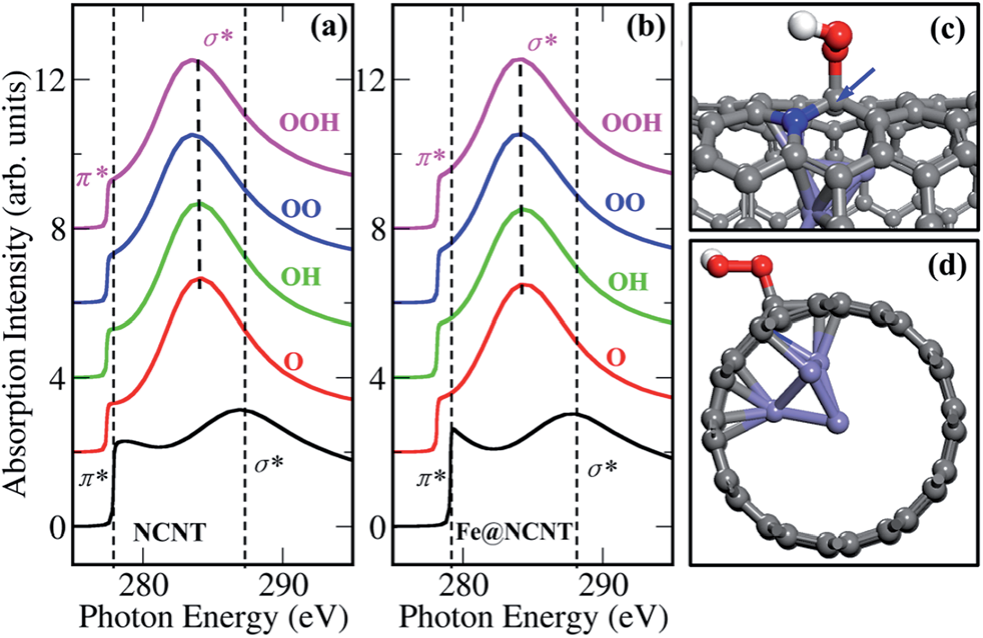
Calculated X-ray absorption spectra at K-edge of carbon in the vicinity of encapsulated Fe_4_ cluster for a bare SWCNT (black curves) and for the same SWCNT with adsorbed oxygen-containing species (colored curves). (a) NCNT, (b) Fe@NCNT, (c) top and (d) side views of Fe@NCNT with an adsorbed OOH species; grey: carbon, red: oxygen, blue: nitrogen, and light-blue: iron.

Thus, we calculated the K-edge of the carbon in the presence of adsorbed oxygen-containing species (O, OH, OO and OOH) on NCNT, as shown in Fig. 5. In comparison with a bare NCNT, the σ* bond position of the same NCNT with adsorbed oxygen-containing species significantly shifts to lower energy (∼285 eV) (Fig. 5a). In addition, the NCNT with an encapsulated iron particle (Fe@NCNT) shows the same feature, *i.e.* the σ* bond position shifts to lower energy (Fig. 5b). Therefore, we are able to understand that the weak signals between π* and σ* bonds observed in measured spectra at ∼288.5 eV, which should originate from the adsorbed oxygen-containing species. Moreover, the pre-edge of π* bond for a Fe@NCNT decorated by oxygen-containing species shifts by ∼1 eV compared with a bare Fe@NCNT. This feature can explain well the increased pre-edge signals observed at ∼283.5 eV (Fig. 2a). By increasing the thickness of the CNT, it was found that the interactions between the outer carbon and the encapsulated iron particle are gradually weakened, as shown in Fig. S9 and S10.† Thus, the chemical origin of the pre-edge signals of carbon can be ascribed to the electronic interactions between carbon shells and encapsulated iron particles, consequently enhanced by the adsorbed oxygen-containing species. These findings explain well our previous work that the encapsulated iron can promote the ORR activity of CNT.^7^ Due to the electron transfers from the enclosed Fe particles to the carbon shells,^7,17^ the local work function of the CNT at iron particle regions decreased which promotes the catalytic activation of O_2_ molecule on carbon shells, thereby the π* signals at this region shift to lower energy at ~283.5 eV (Fig. 2). In a word, the simultaneously measured images and spectra with STXM in this work provide direct evidence of electronic interactions between encapsulated iron particles and carbon shells.

## Conclusion

In summary, STXM has been performed to investigate the electronic structures of CNT with encapsulated iron particles. The chemical images and spectra can directly reveal the electronic interactions between carbon shells and encapsulated iron particles. The enhanced signals of the carbon K-edge at the iron particles regions provide a direct evidence that the iron particles can significantly tune the π* and σ* of carbon shells. A part of electrons are transferred from iron particles to carbon shells, subsequently improving the adsorption of oxygen-containing species, consequently enhancing the signals at pre-edge of C 1s. These characteristics are further confirmed by computed XANES spectra. The present work provides the direct images and spectral evidence of the interactions between carbon shells and encapsulated iron particles, which can promote the understanding towards the interaction of the active sites with support in heterogeneous catalysis.
