# Supplementary material for: Visualizing electronic interactions between iron and carbon by X-ray chemical imaging and spectroscopy
Source: Chem Sci. 2015 Mar 26;6(5):3262–7. doi: 10.1039/c5sc00353a (PMC5490425; doi:10.1039/c5sc00353a)
Supplement: Supplementary file 1 [file SC-006-C5SC00353A-s001.pdf]

Electronic Supplementary Material (ESI) for Chemical Science.
This journal is © The Royal Society of Chemistry 2015

Supplementary Information for

Visualizing electronic interactions between iron and carbon by X-ray chemical imaging and spectroscopy

Xiaoqi Chen[a,ǂ], Jianping Xiao[a,ǂ], Jian Wang[b], Dehui Deng[a,*], Yongfeng Hu[b], Jigang Zhou[b], Liang Yu[a], Thomas Heine[c], Xiulian Pan[a] and Xinhe Bao[a,*]

[a]State Key Laboratory of Catalysis, *i*ChEM, Dalian Institute of Chemical Physics, Chinese Academy of Sciences, Zhongshan Road 457, Dalian, 116023, China.

[b]Canadian Light Source Inc., University of Saskatchewan, 44 Innovation Boulevard, Saskatoon, SK, S7N 2V3, Canada.

[c]Department of Physics and Earth Science, Jacobs University Bremen, Campus Ring 1, 28759 Bremen, Germany.

[ǂ]These authors have contributed equally.

[*]E-mail: dhdeng@dicp.ac.cn; xhbao@dicp.ac.cn;
Fax: +86-411-84379128; Tel: +86-411-84686637.

Experimental Section:

Synthesis of Pod-Fe: The synthesis process is the same as our previous report.[1] Briefly, 3.0 g ammonium hexacyanoferrate(II) hydrate($(NH_4)_4Fe(CN)_6\cdot xH_2O$) (Alfa Aesar) was heated at 600 °C for 2 h in Ar with a flow rate of 30 mL/min. The obtained sample was treated in 1.0 M H_2SO_4 aqueous solution at 90°C for 4 h to remove exposed iron. The product was then washed in distilled water and ethanol, and drying at 120 °C for 12 h. The obtained sample was denoted as Pod-Fe.

STXM Measurement: The Pod-Fe powder samples were dispersed in methanol by ultrasound to form a homogeneous suspension. Then a droplet of the sample suspension was deposited onto a blank Si_3N_4 window using a micropipette and dried in the air. STXM was conducted at the Soft X-ray Spectromicroscopy (SM) 10ID-1 beamline of the Canadian Light Source. In STXM, the monochromatic X-ray beam is focused by a Fresnel zone plate to a ~30 nm spot on the sample, and the sample is raster-scanned with synchronized detection of transmitted X-rays to generate image sequences (stacks) over a range of photon energies. Circular polarization was used to remove any dichroic effect for CNT. STXM data were analyzed by aXis2000 (available free for noncommercial applications at

http://unicorn.mcmaster.ca/aXis2000.html). The C, N, O K-edges, and Fe L-edge image stacks were appended together to form one whole stack, and then aligned and converted to absorbance (i.e. optical density). XANES (X-ray absorption near edge structure) was extracted from the combined image stack using an image mask. Chemical imaging was conducted by image average and subtraction on the absorption edge/XANES features. Chemical composition of the sample was determined by fitting the multi-edge absolute X-ray absorption (OD) spectra extracted from STXM image stacks with the atomic mass X-ray absorption coefficients and the estimated density of the sample in aXis2000.[2, 3] The sample density was estimated based on the density of graphite and metallic iron, and the fact that covalent atomic size of C, N and O is comparable.

Powder sample XANES measurement: XANES measurements of powder samples were performed at High Resolution Spherical Grating Monochromator (SGM) 11ID-1 beamline of the Canadian Light Source using a total electron yield mode (TEY) and fluorescence yield mode (FY).

Transmission electron microscopy (TEM) measurement: TEM measurement was carried out on a FEI Tecnai G2 microscope operated at an accelerating voltage of 120 kV.

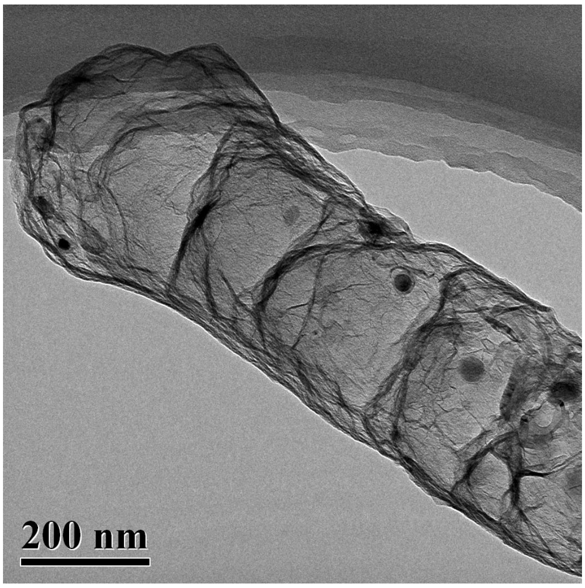

Fig. S1 TEM image of Pod-Fe, showing bean pod-like structure of carbon with iron particles encapsulated in the compartments.

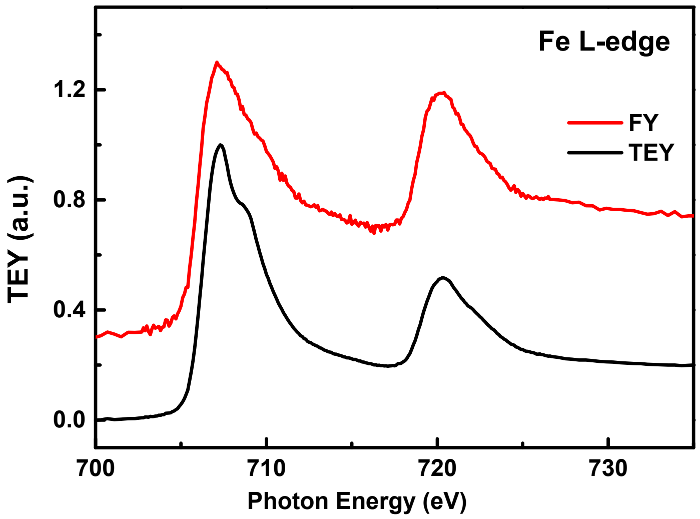

Fig. S2 Fe L-edge XANES spectra of Pod-Fe powder sample measured at TEY and FY mode simultaneously.

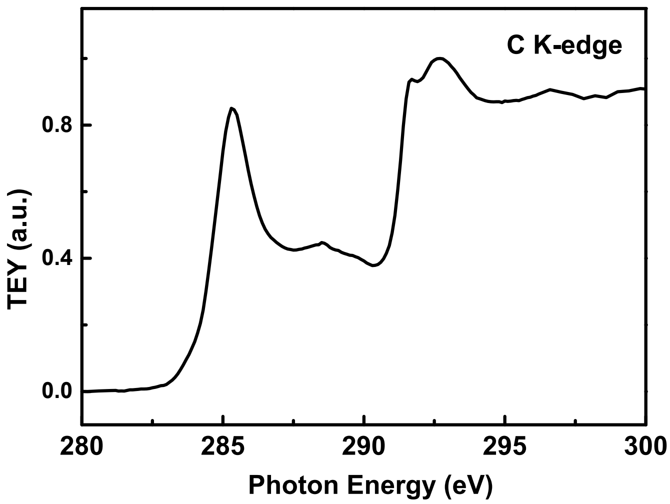

Fig. S3 C K-edge XANES spectra of Pod-Fe powder sample measured at TEY mode.

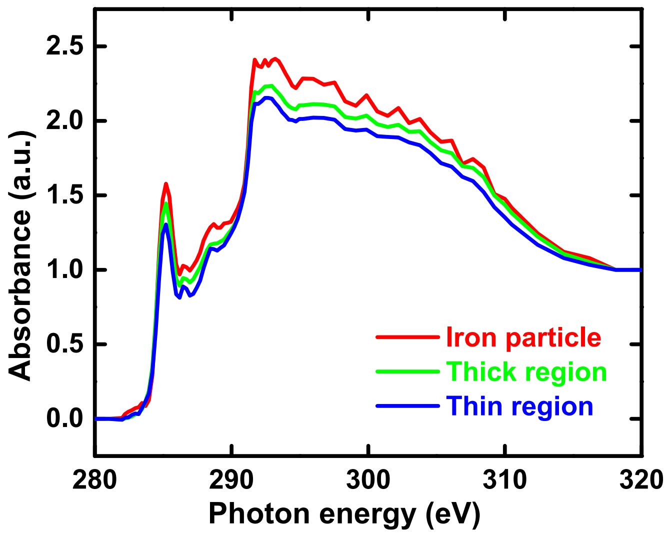

Fig. S4 C K-edge XANES spectra of Pod-Fe of STXM normalized from pre-edge and post-edge.

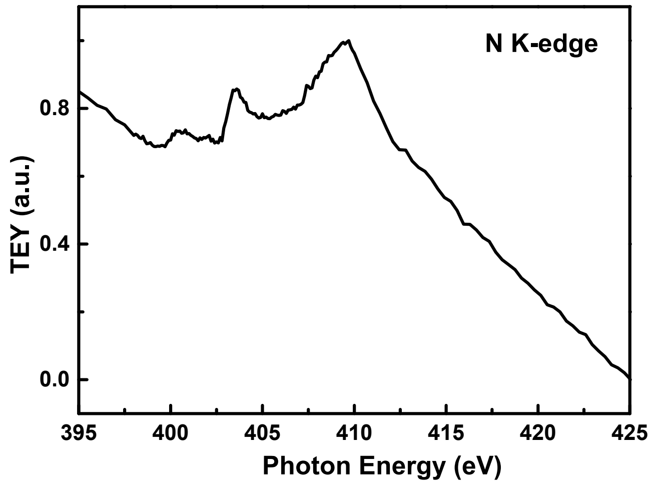

Fig. S5 N K-edge XANES spectra of Pod-Fe powder sample measured at TEY mode. The signal shows that there is some amount of nitrogen doping in Pod-Fe.

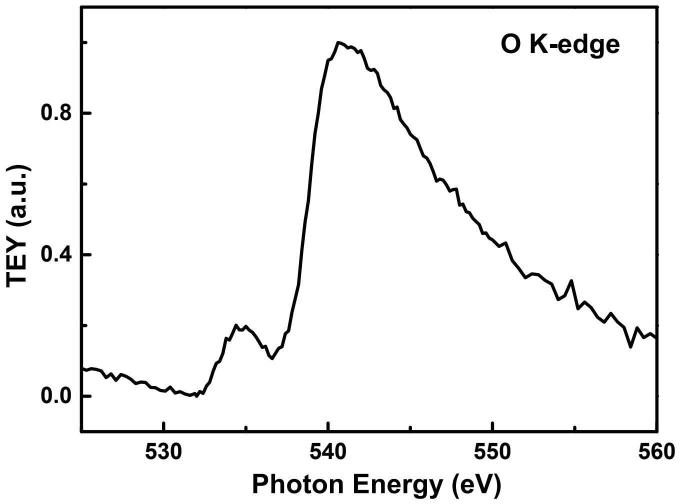

Fig. S6 O K-edge XANES spectra of Pod-Fe powder sample measured at TEY mode.

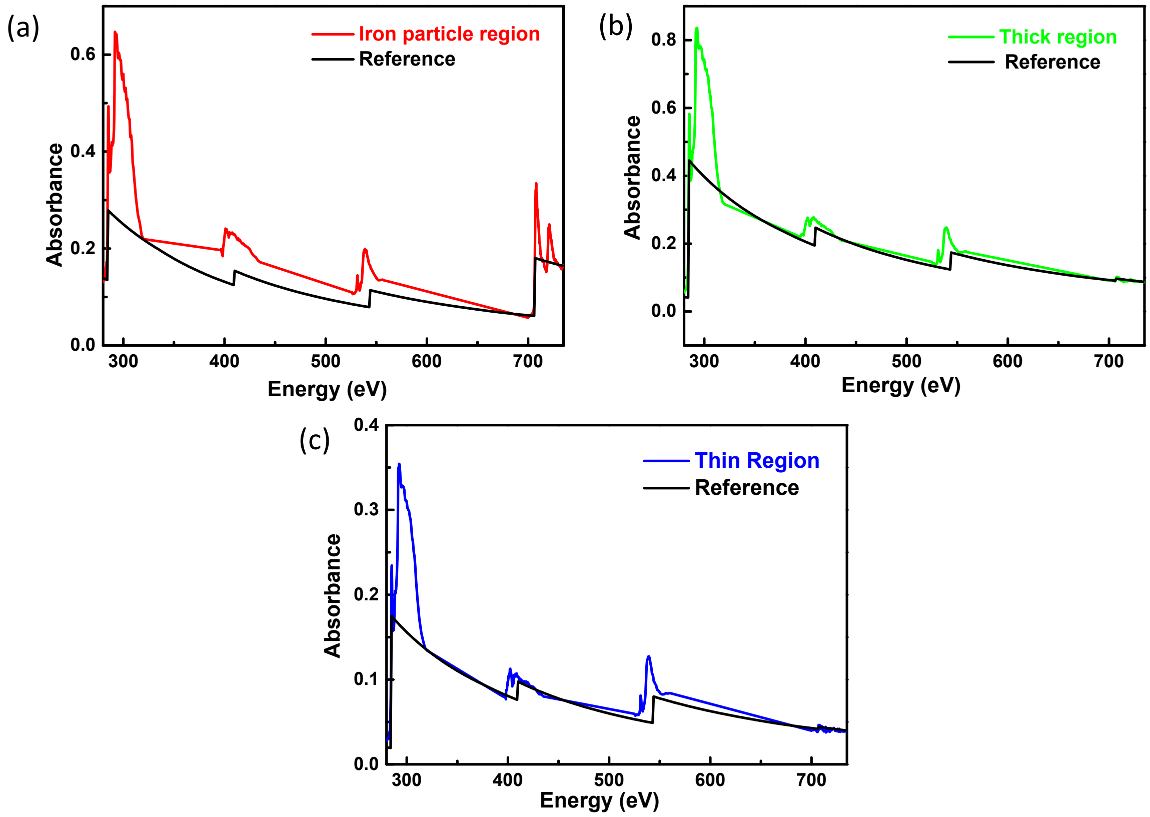

Fig. S7 Element fit to calculated pure atomic X-ray absorption reference spectra from averaged spectra from the relevant sample regions: (a) Iron particle region; (b) thick CNT region; (c) thin CNT region.

Table. S1 Estimated chemical composition from multi-edge absolute X-ray absorbance (OD) spectra extracted from STXM stacks. For chemical formula, carbon is treated as "1", and others are relative to carbon.

Chemical Composition		C	N	O	Fe	Estimated Density (g/cm^3)[a]	Thickness (nm)[b]
Iron particle region	Formula	1	0.30	0.45	0.55	3.71	35.5
	Mass (%)	22.1%	7.7%	13.3%	56.8%		
Thick CNT region	Formula	1	0.19	0.23	0.01	2.36	54.5
	Mass (%)	63.5%	14.1%	19.5%	3.0%		
Thin CNT region	Formula	1	0.20	0.37	0.01	2.41	23.0
	Mass (%)	56.4%	13.2%	27.8%	2.6%		
Graphite	Formula	1	-	-	-	2.16	-
Metallic Iron	Formula	-	-	-	1	7.87	-

[a]Density of graphite and metallic iron is real; other densities are estimated based on the density of graphite and metallic iron, and comparable covalent atomic size of C, N and O.

[b]Note that the sample thickness is roughly estimated by fitting the multi-edge absolute X-ray absorption (OD) based on the database.[2, 3]

Computational details:

XANES calculations were performed by using cluster models, in which the local geometries of all simulated structures are optimized by DFT calculations from our previous works.[1, 4] The models of single-walled carbon nanotube (SWCNT), double-walled carbon nanotube (DWCNT), and triple-walled carbon nanotube (TWCNT) are constructed with CNT(6,6), CNT(6,6)@CNT(11,11), CNT(6,6)@CNT(11,11)@CNT(16,16), respectively, and an encapsulated Fe_4 cluster. The Hedin-Lundquist exchange functional was employed in all calculations and the core-hole effect was also corrected by random-phase approximation screened scheme. The E-vector was taken from the average of all orientations in the calculations.

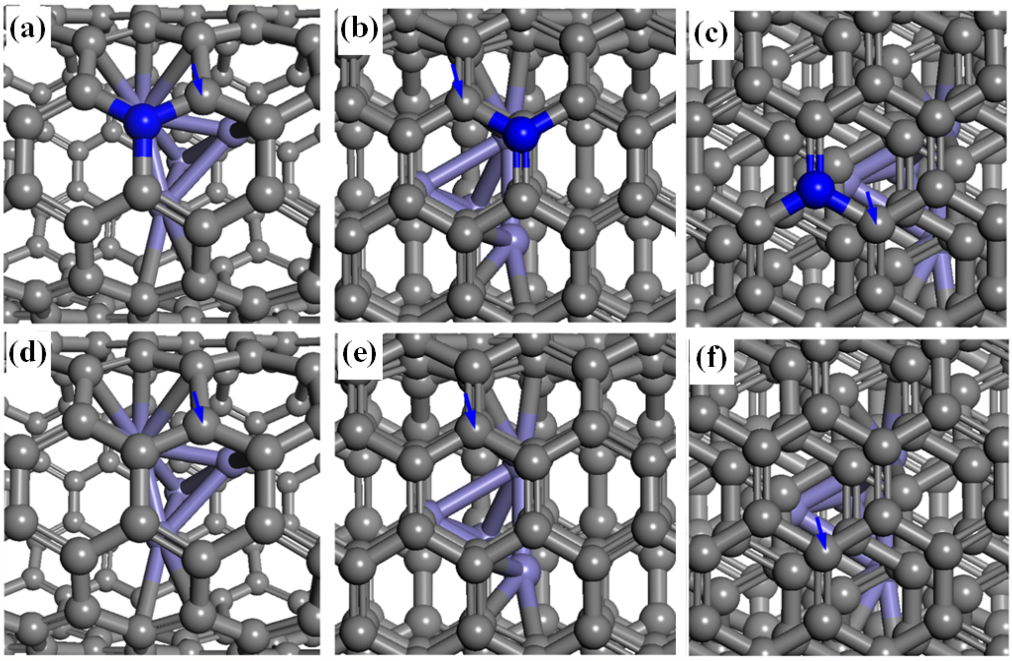

Fig. S8 Atomistic models of (a) a SWCNT, (b) a DWCNT, and (c) a TWCNT with a encapsulated Fe_4 cluster and a doped nitrogen atom; (d) a SWCNT, (e) a DWCNT, and (f) a TWCNT with a encapsulated Fe_4 cluster in XANES calculations with the calculated carbon indicated by blue arrow in all models. Grey: carbon, blue: nitrogen, and light-blue: iron. The blue arrow marks the active site for the adsorption of oxygen species.

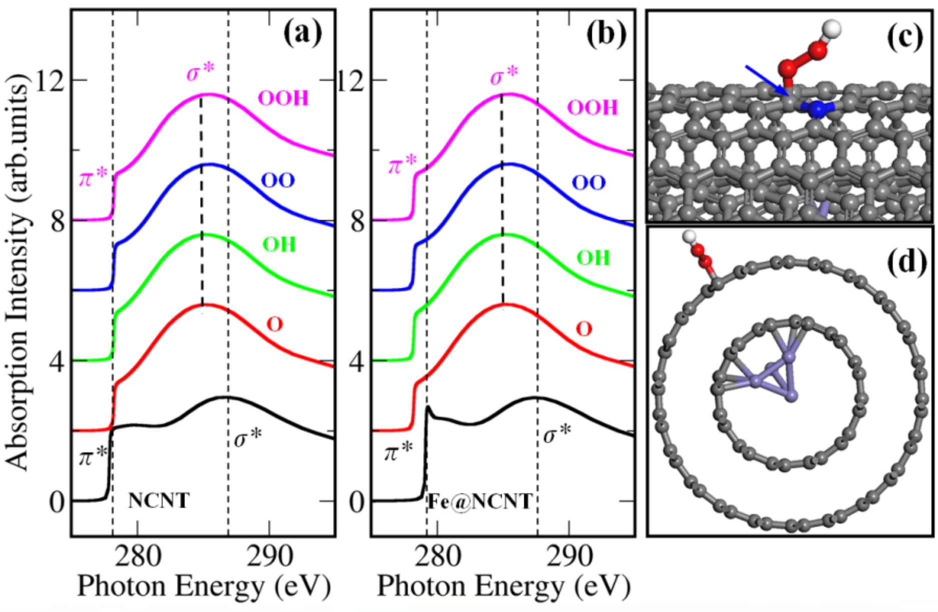

Fig. S9 Calculated X-ray absorption near K-edge spectra of carbon shells in the vicinity of encapsulated Fe_4 cluster for a bare DWCNT (black curves) and the same DWCNT with adsorbed oxygen-containing groups (color curves); (a) NCNT, (b) Fe@NCNT, (c) top and (d) side views of Fe@NCNT with an adsorbed OOH species (Grey: carbon, red: oxygen, blue: nitrogen, and light-blue: iron.). The calculated carbon is indicated by blue arrow.

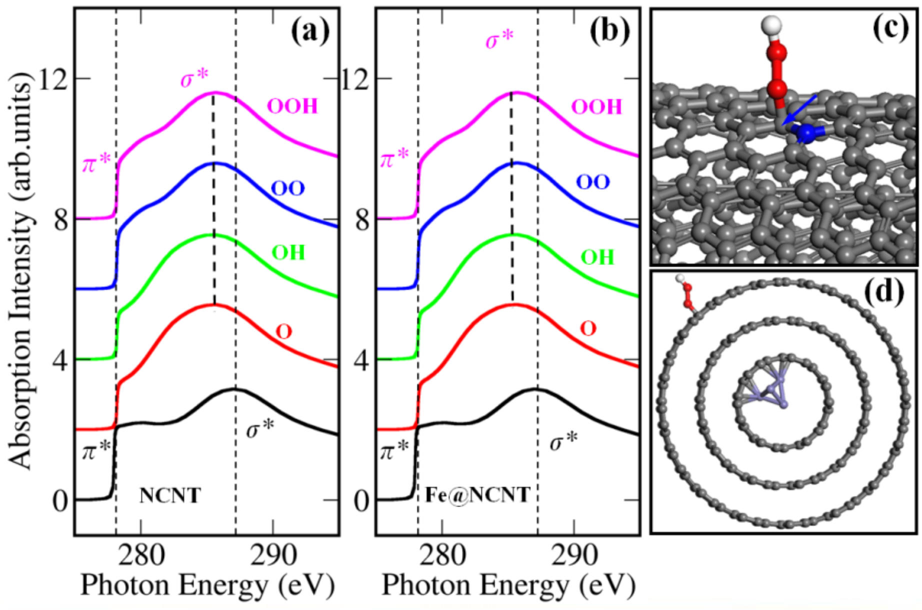

Fig. S10 Calculated X-ray absorption near K-edge spectra of carbon atoms in the vicinity of encapsulated Fe_4 cluster for a bare TWCNT (black curves) and the same TWCNT with adsorbed oxygen-containing groups (color curves). (a) NCNT, (b) Fe@NCNT, (c) top and (d) side views of Fe@NCNT with an adsorbed OOH species (Grey: carbon, red: oxygen, blue: nitrogen, and light-blue: iron.). The calculated carbon is indicated by blue arrow.

Reference:

1. D. Deng, L. Yu, X. Chen, G. Wang, L. Jin, X. Pan, J. Deng, G. Sun and X. Bao, *Angew. Chem., Int. Ed.*, 2013, **52**, 371-375.
2. B. L. Henke, E. M. Gullikson and J. C. Davis, *At. Data Nucl. Data Tables*, 1993, **54**, 181-342.
3. *http://henke.lbl.gov/optical_constants/filter2.html*.
4. J. Deng, L. Yu, D. Deng, X. Chen, F. Yang and X. Bao, *J. Mater. Chem. A*, 2013, **1**, 14868-14873.
